# TET-Mediated Epigenetic Regulation in Immune Cell Development and Disease

**DOI:** 10.3389/fcell.2020.623948

**Published:** 2021-01-15

**Authors:** Nikolas James Tsiouplis, David Wesley Bailey, Lilly Felicia Chiou, Fiona Jane Wissink, Ageliki Tsagaratou

**Affiliations:** ^1^University of North Carolina Lineberger Comprehensive Cancer Center, Chapel Hill, NC, United States; ^2^University of North Carolina Center of Translational Immunology, Chapel Hill, NC, United States; ^3^University of North Carolina Institute of Inflammatory Disease, Chapel Hill, NC, United States; ^4^University of North Carolina Curriculum in Genetics and Molecular Biology, Chapel Hill, NC, United States; ^5^University of North Carolina Department of Genetics, Chapel Hill, NC, United States; ^6^University of North Carolina Department of Microbiology and Immunology, Chapel Hill, NC, United States

**Keywords:** TET proteins, epigenetics, 5hmC, immune cell development, cancer

## Abstract

TET proteins oxidize 5-methylcytosine (5mC) to 5-hydroxymethylcytosine (5hmC) and further oxidation products in DNA. The oxidized methylcytosines (oxi-mCs) facilitate DNA demethylation and are also novel epigenetic marks. TET loss-of-function is strongly associated with cancer; *TET2* loss-of-function mutations are frequently observed in hematological malignancies that are resistant to conventional therapies. Importantly, TET proteins govern cell fate decisions during development of various cell types by activating a cell-specific gene expression program. In this review, we seek to provide a conceptual framework of the mechanisms that fine tune TET activity. Then, we specifically focus on the multifaceted roles of TET proteins in regulating gene expression in immune cell development, function, and disease.

## Introduction

Gene expression in mammalian cells is a highly regulated process whereby transcription factors (TFs) bind to specific DNA-binding motifs within promoters and enhancers in distinct cell types, causing them to differentiate and acquire new cell fates (Roadmap Epigenomics Consortium et al., 2015). These processes are both spatially and temporally specific, resulting in the varied assortment of cell types observed in mammals. While some of the TFs are ubiquitously expressed, others exhibit a cell-specific expression pattern. In some cases, the same TF can regulate different genes in different cells, highlighting the dynamic nature of regulatory networks across the organism (Lambert et al., 2018). Epigenetic markers provide an additional component of regulation to this process by modifying the accessibility of the histones surrounding DNA, or even the DNA itself (Bernstein et al., 2007). Two of the primary epigenetic modifications are histone post-translational modifications (Zhou et al., 2011) and DNA methylation (Smith and Meissner, 2013). In mammalian cells, transcription of the vast majority of protein-coding genes starts at promoters, which are rich in CG sequences (Bogdanovic and Lister, 2017). DNA methylation occurs on cytosine bases within CpG islands (Lister et al., 2009). DNA methylation of cytosine involves the covalent addition of a methyl group at position 5 of the pyrimidine ring of cytosine and is achieved by the catalytic activity of the family of DNA methyltransferases (DNMTs) (Goll and Bestor, 2005), which consists of DNMT1, DNMT2, DNMT3a, and DNMT3b. In the human genome, 60–80% of 28 million CpG dinucleotides are methylated (Lister et al., 2009; Ziller et al., 2013).

Genome-wide studies using bisulfite sequencing to assess cytosine methylation have established that highly transcribed genes have sparsely methylated CpG promoters, whereas silenced, non-transcribed genes show high levels of cytosine methylation in the CpG context of their promoters (Lister et al., 2009; Laurent et al., 2010). Methylation of repetitive DNA sequences, found close to centromeres, is instrumental in the maintenance of genomic integrity. In mice, repetitive DNA can be distinguished in major satellites (243 bp repeat sequences) found in the pericentromeric region as well as in minor satellites (120 bp repeat sequences) found in the centromeric region (Guenatri et al., 2004). Aberrations in DNA demethylation are a hallmark of cancer and can result in silencing of tumor suppressor genes by increasing the methylated cytosines at their promoters. Conversely, global hypomethylation leads to genomic instability (Baylin and Jones, 2011).

**Figure 1:**
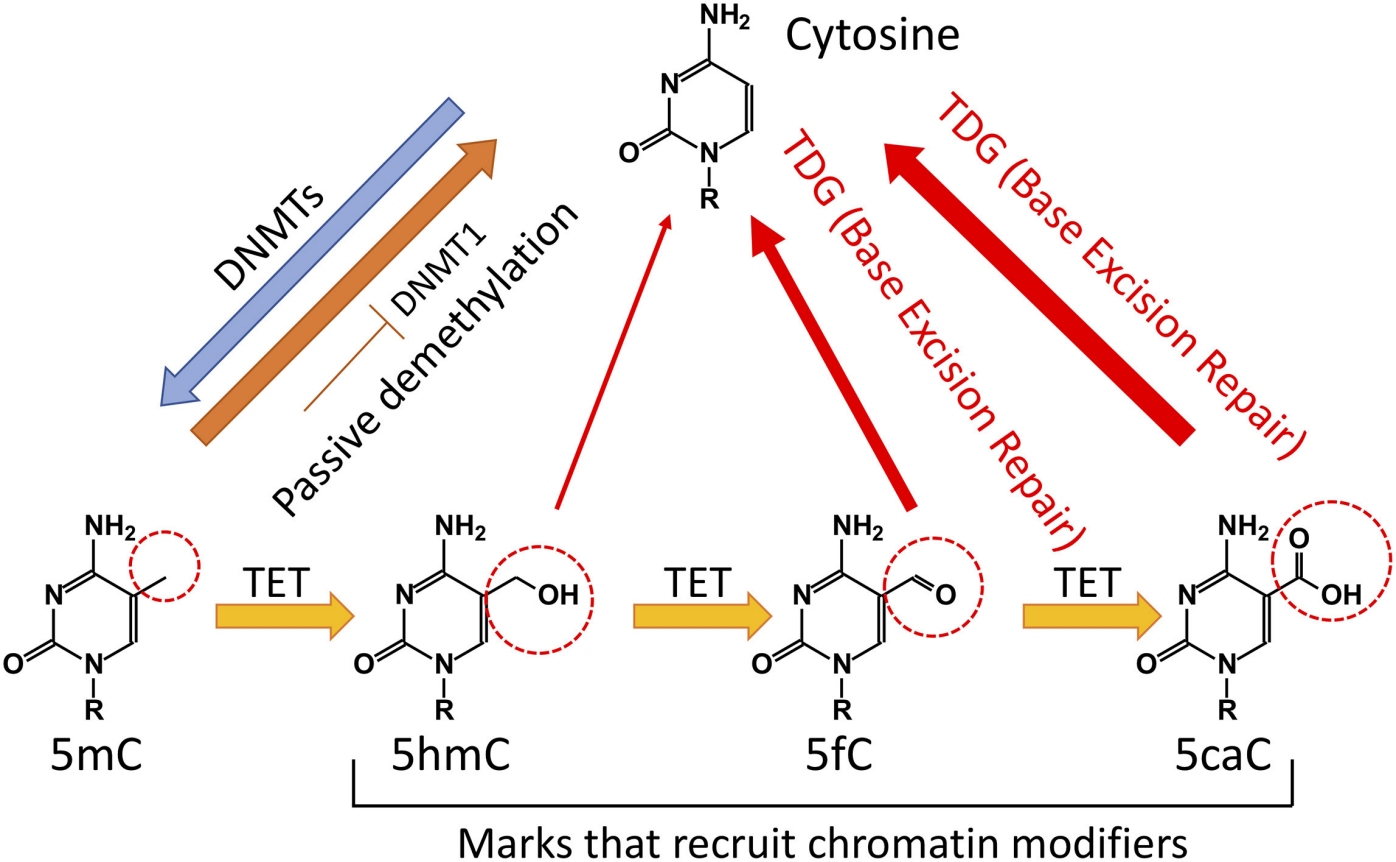
Regulation of DNA methylation in mammalian cells. Cytosine (C) is methylated by DNA methyltransferases (DNMTs) to 5-methylcytosine (5mC). Cytosine demethylation can occur in the absence of enzymatic activity during cell division. In addition, Ten Eleven Translocation (TET) proteins can oxidize 5mC to 5-hydroxymethylcytosine (5hmC). A significant portion of 5hmC will be diluted during cell division. TET proteins can further oxidize 5hmC to 5-formylcytosine (5fC) and 5-carboxylcytosine (5caC). The TDG through the Base Excision Repair (BER) can convert 5fC and 5caC to unmodified C.

Previously, it was believed that DNA methylation was an irreversible event that could only be removed passively *via* dilution during DNA replication. However, the Ten Eleven Translocation (TET) family of proteins has been shown to catalyze the subsequent oxidations of 5-methylcytosine (5mC) to 5-hydroxymethylcytosine (5hmC), 5-formylcytosine (5fC), and 5-carboxylcytosine (5caC) (Tahiliani et al., 2009; He et al., 2011; Ito et al., 2011) (Figure 1). TET proteins therefore provide an active pathway for DNA demethylation and consequently have relevance for regulation of gene expression. TET proteins mediate “active” (replication-independent) DNA demethylation *via* excision of 5fC and 5caC by thymine DNA glycosylase (TDG). Afterwards, base excision repair machinery substitutes the excised base with an unmethylated cytosine (Branco et al., 2012; Pastor et al., 2013). Notably, the majority of 5hmC is passively diluted *via* replication (Tsagaratou et al., 2014; Nestor et al., 2015) (Figure 1).

Indicating a conserved role in controlling DNA demethylation, representatives of the TET/JBP superfamily have been reported in every metazoan organism (Iyer et al., 2009; Pastor et al., 2013). In mammalian cells specifically, there are three TET proteins: TET1, TET2, and TET3. TET1 was identified as a fusion partner of the mixed-lineage leukemia (MLL) gene from the breakpoint of chromosomal translocation t(10;11)(q22;q23) in acute myeloid leukemia (AML) (Lorsbach et al., 2003). Studying mouse models over the life course has shown that TET1 and TET2 are most highly expressed in the inner cell mass and embryonic stem (ES) cells (Tahiliani et al., 2009; Koh et al., 2011). TET2 is expressed at lower levels than TET1 in ES cells, and its expression first drops and then increases upon differentiation; it is expressed in numerous differentiated organs and cell types in the adult (Pastor et al., 2013; Tsagaratou and Rao, 2014). TET1 is also highly expressed in primordial germ cells (PGCs) (Hackett et al., 2013; Vincent et al., 2013), while TET2 and TET3 are highly expressed throughout the remainder of development. TET3 exhibits high expression in oocytes and zygotes (Gu et al., 2011), and loss of TET3 in mice results in perinatal lethality (Pastor et al., 2013). Both TET1 and TET2 are implicated in cancer. TET1 is an MLL partner in cases of acute myeloid (AML) and lymphoid (ALL) leukemias, while loss of function of TET2 is strongly associated with myelodysplastic syndromes, myeloproliferative neoplasms, and myeloid leukemias (Ko et al., 2010).

**Figure 2:**
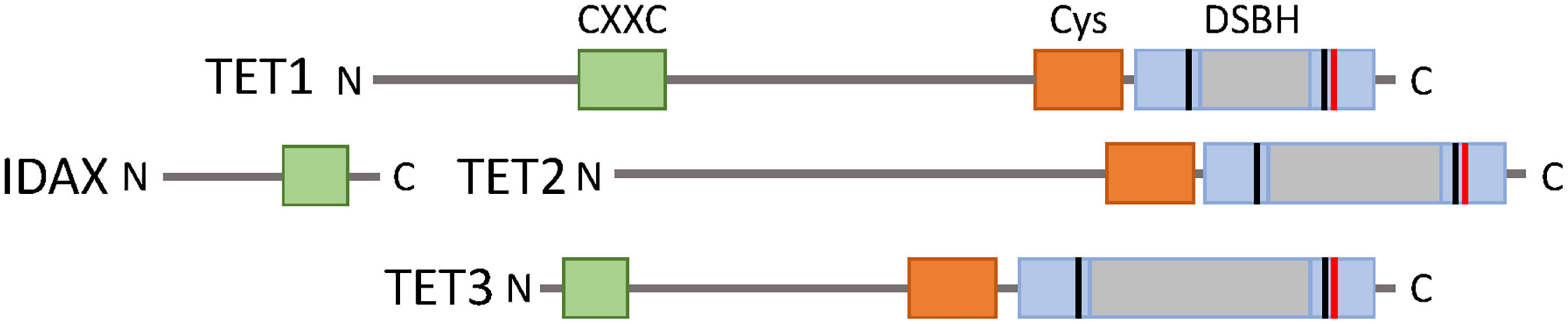
The TET family of proteins. TET1, TET2, and TET3 share a C-terminal catalytic domain consisting of cysteine-rich (orange) and double stranded β-helix (gray) domains, and binding sites for cofactors Fe(II) (black) and 2-oxoglutarate (red). TET1 and TET3 have an N-terminal CXXC DNA binding domain, but this was lost in TET2 from a chromosomal inversion and became a separate protein IDAX.

TET proteins arose from a common ancestral gene that underwent triplication in jawed vertebrates. TET1 and TET3 have greater structural similarities, as they share an N-terminal CXXC DNA binding domain. However, TET2 lacks a CXXC domain and thus cannot directly bind to DNA. During evolution, the ancestral *Tet2* gene underwent a chromosomal inversion that resulted in separation of the TET2 CXXC DNA binding domain from the rest of the protein. The CXXC DNA binding domain of TET2 became a separate gene known as IDAX (Iyer et al., 2009; Ko et al., 2013) (Figure 2). The core catalytic domain on each TET protein is comprised of a cysteine-rich domain, a conserved double-stranded β-helix (DSBH) domain, and binding sites for the cofactors α-ketoglutarate (α-KG) and Fe (II) (Pastor et al., 2013). Studies have indicated that these catalytic domains preferentially bind to cytosines on CpG islands without interacting with adjacent bases (Pastor et al., 2013) (Figure 2).

5hmC is found at different levels in mammalian cells. It is most abundant in Purkinje neurons, where it comprises ~40% of 5mC levels (Kriaucionis and Heintz, 2009). In ES cells, the levels of 5hmC vary between 5 and 10% of the levels of 5mC, whereas it is present at only 1% of the total level of 5mC in some immune populations (Ko et al., 2010). 5hmC in gene bodies and enhancers has been positively correlated with increased gene expression in various cell types such as neural cells (Mellen et al., 2012; Lister et al., 2013), T cells (Tsagaratou et al., 2014; Ichiyama et al., 2015), B cells (Lio et al., 2016; Orlanski et al., 2016), and spermatogenic cells (Gan et al., 2013). TET-mediated DNA demethylation in distal enhancers occurs at a higher rate than passive demethylation (Ginno et al., 2020). 5fC and 5caC are even less abundant compared to 5hmC (He et al., 2011). In addition to their role in mediating DNA demethylation, the oxidative derivatives of TET function−5hmC, 5fC, and 5caC—are also stable epigenetic marks (Bachman et al., 2014, 2015) that can be specifically recognized and preferentially bound by readers—mainly transcriptional regulators—to impact transcriptional elongation, genomic integrity, and DNA repair (Yildirim et al., 2011; Mellen et al., 2012; Iurlaro et al., 2013; Spruijt et al., 2013; Hashimoto et al., 2014; Xiong et al., 2016; Chen et al., 2017; Cimmino and Aifantis, 2017; Tsagaratou et al., 2017a; Wu and Zhang, 2017; Parry et al., 2020; Shukla et al., 2020) (Figure 1). For instance, Ubiquitin-like protein containing PHD and RING finger domains 1 (UHRF1) as well as methyl-CpG-binding protein 2 (MeCP2) can bind 5mC and 5hmC (Frauer et al., 2011; Mellen et al., 2012). It was suggested that 5hmC binding by MeCP2 in neural cells alters chromatin structure and facilitates gene expression (Mellen et al., 2012). Experiments in mESCs revealed that 5fC and 5caC are involved in specific binding interactions with a greater number of proteins in comparison to 5hmC (Spruijt et al., 2013). During their cell cycle-independent removal by the base excision repair pathway, 5fC and 5caC recruit an increased number of DNA repair proteins compared to 5hmC (Spruijt et al., 2013). Moreover, 5caC can be recognized by basic helix-loop-helix proteins such as MAX and TCF4 (Wang et al., 2017; Yang et al., 2019) as well as Wilms tumor protein (Hashimoto et al., 2014).

In addition to promoting binding of some transcription factors, modified cytosines can inhibit binding of transcription factors and transcriptional activators to suppress gene expression. For instance, the presence of 5hmC within the sequence of a cAMP response element (CRE) at an artificial promoter can decrease the binding of the transcriptional activator c-AMP Response Element Binding (CREB) protein, resulting in decreased expression of the target genes (Kitsera et al., 2017). On the other hand, oxi-mCs can also prevent binding of transcriptional repressors and thus promote gene expression. MDB1 can specifically bind to 5mC but not to oxi-mCs and recruit the histone methyltransferase SETDB1 to promote H3K9 methylation and repress the expression of proopiomelanocortin (*Pomc*) (Marco et al., 2016). Thus, the presence of oxi-mCs prevents binding of SETDB1 and promotes the *Pomc* expression.

In the next sections we will first summarize the known mechanisms that regulate TET function. Although the majority of the described mechanistic studies have been performed in embryonic stem cells or cell lines, we should note that these mechanisms might be applicable to an array of other cell types, including immune cells, the major focus of this review. Then, we will discuss in detail how TET proteins shape immune cell development and function.

## Mechanisms of TET Function

### Competition Among TET Proteins and DNMTs

Mammalian genomes maintain high levels of CpG methylation (Lister et al., 2009; Ziller et al., 2013) even though the enzymes that regulate DNA methylation, DNMTs, and TET proteins are concomitantly expressed. Various studies have suggested a dynamic regulation of DNA methylation that is achieved through focal competition between TET proteins and DNMTs in pluripotent cells. Bivalent promoters are marked simultaneously by H3K4me3 and H3K27Ac but exhibit low levels of DNA methylation (Mikkelsen et al., 2007). However, loss of TET1, TET2, and TET3 resulted in aberrant hypermethylation of bivalent promoters given that DNMT3B could act without any competition from TET proteins on these genomic loci (Verma et al., 2018). As a result, the expression of lineage-specifying transcription factors was prohibited, and proper cellular differentiation was hindered (Verma et al., 2018). TET1-specific chromatin immunoprecipitation followed by sequencing (ChIP-seq) experiments in mESCs revealed that TET1 binds to bivalent promoters (Xu et al., 2011). TET1 seems to exclude DNMT3A1, the longer isoform of DNMT3A, from proximal promoters and canyons where TET1 seems to preferentially bind in embryonic stem cells (Gu et al., 2018). Moreover, it was shown that TET proteins compete with DNMT proteins to regulate the methylation status of enhancers (Verma et al., 2018; Charlton et al., 2020).

### TET Proteins and Interacting Partners

TET proteins mediate a cell-specific, focal DNA demethylation. This is broadly achieved by interaction with transcription factors that mediate the recruitment of TET proteins onto the DNA. For instance, SALL4 in enhancers of mouse embryonic stem cells (ESCs) interacts with TET1 and binds to 5hmC (Xiong et al., 2016). Subsequently, SALL4 mediates the recruitment of TET2 that further oxidizes 5hmC (Xiong et al., 2016). This stepwise oxidation of 5mC to other oxi-mCs tightly regulates gene expression of developmental genes in mouse ESCs (Xiong et al., 2016). In addition, TET1 and TET2 can interact with Nanog to enhance reprogramming efficiency in a catalytic-dependent manner (Costa et al., 2013). RINF (also known as CXXC5) can form a complex with NANOG, OCT4, TET1, and TET2, facilitating the recruitment of the complex to the DNA; RINF also regulates the expression of TET proteins (Ravichandran et al., 2019).

TET proteins interact with various heterochromatin-associated proteins such as SIN3A, HDAC1, and HDAC2 (Ficz et al., 2011; Williams et al., 2011). This can affect chromatin modifications and ultimately impact gene expression. For example, TET proteins interact with OGlcN-Acetyl Transferase (OGT), subsequently impacting histone modifications and gene expression (Chen et al., 2013; Deplus et al., 2013). TET interaction with OGT can also impact TET protein stability (Shi et al., 2013) and activity (Vella et al., 2013).

Moreover, TET proteins interact with components of the Base Excision Repair Complex (BER), such as PARP1, LIG3, and XRCC1 (Muller et al., 2014), as well as DNA glycosylases, such as Thymine DNA glycosylase, NEIL, and MBD4 (Muller et al., 2014), therefore suggesting a role in DNA repair. 5hmC has been found to be increased in cells upon treatment with DNA-damaging agents in cell lines (Kafer et al., 2016). Deletion of TET1 results in increased accumulation of DNA breaks as evaluated by increased staining for γH2Ax (Kafer et al., 2016).

### Post-translational Modifications and DNA Binding

How the tri-dimensional structure of TET proteins is controlled remains less understood. Recent studies in the past years revealed that TET proteins are post-translationally modified. The interaction with OGT results in O-GlcNAcylation of TET1 and TET2 in ESCs (Vella et al., 2013). In addition, all three TET proteins can be phosphorylated (Bauer et al., 2015) at their N-terminus as well as at the low-complexity insert region between the two parts responsible for dioxygenase activity. Interestingly, there seems to be competition between O-GlcNAcylation and phosphorylation. Indeed, some peptides have both post-translational modifications (PTMs) (Bauer et al., 2015). These protein sequences within TET proteins could act as PTM switches that influence the PTM pattern on the neighboring amino acid (Bauer et al., 2015). For TET2 and TET3, a variety of PTMs are observed in highly modified regions. In the case of TET2, phosphorylation and O-GlcNacylation do not co-occur at the same amino acid. For TET3, however, the same amino acid could have both PTMs in some cases. PTMS in TET1 were more isolated. This dynamic interplay of phosphorylation and O-GlcNAcylation could facilitate dynamic changes in TET protein localization, activity, or targeting to genomic loci in response to external stimuli or environmental cues (Bauer et al., 2015).

AMP-activated protein kinase (AMPK) can phosphorylate murine TET2 at the serine residue 97 (Wu et al., 2018; Zhang et al., 2019). This phosphorylation event stabilizes TET2 (Wu et al., 2018; Zhang et al., 2019) which can then demethylate enhancers as C2C12 cells differentiate to myotubes (Zhang et al., 2019).

Moreover, it has been reported that TET conformation and DNA-binding ability can be affected by ubiquitination (Nakagawa et al., 2015). Specifically, VprBP interacts with TET2 by binding to the C-terminal dioxygenase catalytic domain of TET2. VprBP can also bind to the catalytic domain of TET1 and TET3 (Nakagawa et al., 2015). Notably, deletion of VprBP in mouse embryonic fibroblasts (MEFs) results in reduction of 5hmC, suggesting that VprBP is essential for TET protein function (Nakagawa et al., 2015).

TET2 can be acetylated by p300 at lysine K110 and deacetylated by HDAC1/2 (Zhang et al., 2017). Acetylation increases TET2 activity and stability as well as the interaction of TET2 with DNMT1, which targets TET2 to chromatin (Zhang et al., 2017). Importantly, oxidative stress can target the TET2/DNMT1 complex to chromatin, resulting in elevated DNA methylation and hydroxymethylation (Zhang et al., 2017). Loss of TET2 and subsequent induction of oxidative stress results in aberrant gain of methylation at CGI promoters and enhancers. Acetylation of TET2 can also increase interaction with DNMT3b; however, DNMT3b cannot target TET2 to chromatin as DNMT1 (Zhang et al., 2017). In addition, p300 can acetylate TET1 and TET3, but this most likely occurs in different lysine residues since K110 is not conserved among TET proteins (Zhang et al., 2017). Deacetylation of TET2 results in disassembly from DNMT3, polyubiquitination, and proteasome degradation (Zhang et al., 2017).

### TET Proteins and RNA Modification

5hmC has also been detected in RNA (Delatte et al., 2016; Zhang et al., 2016). It has been reported to preferentially mark polyadenylated RNAs in Drosophila (Delatte et al., 2016). Studies suggest that 5hmC in the RNA can facilitate mRNA translation (Delatte et al., 2016). In addition, TET2 has been shown bind to RNA in mESCs; this is mediated *via* its interaction with the RNA-binding protein Paraspeckle component 1 (PSPC1) (Guallar et al., 2018). TET2 and PSPC1 mediate the silencing of endogenous retroviruses (ERVs). MERVL was among the ERVs that were repressed in the aforementioned studies. It was shown that catalytic activity of TET2 was required for repression (Guallar et al., 2018). The PSPC1 and TET2 complex could bind to both 5mC and 5hmC RNAs but had higher affinity for 5mC-containing RNAs (Guallar et al., 2018). The PSPC1-TET2 mediated 5hmC deposition on MERVL transcripts resulted in their destabilization and subsequent degradation (Guallar et al., 2018). In addition, both TET1 and TET2 deposit 5hmC in mRNAs in genes that are fundamental for pluripotency, such as *Jarid2* and *Eed*, and can result in reduced mRNA stability (Lan et al., 2020). As a consequence of the transcript destabilization, pluripotency genes that would be expressed too highly acquire appropriate expression levels and adequately repress the expression of lineage-specifying factors (Lan et al., 2020). As the ES cells receive differentiation cues, the pluripotency factors are rapidly turned off and the lineage-specifying factors are upregulated to drive the differentiation process with efficiency (Lan et al., 2020).

### TET Proteins and Catalytic-Independent Roles

TET proteins can also regulate gene expression in a catalytic-independent manner *via* interactions with other proteins that affect chromatin architecture and transcription. An example is the formation of the TET1-SIN3A complex (Williams et al., 2011). The SIN3A complex—together with its components, histone deacetylases 1 and 2 (HDAC1/2)—can repress transcription by mediating histone deacetylation. In addition, TET3 can also interact with SIN3A *via* a TET SIN3A interaction domain (SID) that interfaces directly with the paired amphipathic helix (PAH) domain of SIN3A (Deplus et al., 2013; Chandru et al., 2018). The SID domain is necessary for TET1 to suppress gene expression (Chandru et al., 2018) and is not part of its catalytic domain. Interestingly, this domain is present in TET1 and TET3 but not TET2 (Chandru et al., 2018). Furthermore, TET1 was shown to interact with Hypoxia Factor (HIF)−1a and HIF-2a to act as co-activator and promote gene expression in a catalytic-independent manner (Tsai et al., 2014). The CXXC DNA binding domain of TET1 is required for this interaction (Tsai et al., 2014). In addition, TET3 fine-tunes adult neurogenesis in a catalytic-independent manner (Montalban-Loro et al., 2019). TET3 prevents premature differentiation of neural stem cells (NSCs) into astrocytes in the adult subventricular zone by inhibiting the expression of Small nuclear ribonucleoprotein-associated polypeptide (*Snrpn*) (Montalban-Loro et al., 2019). This is achieved by direct binding of TET3 to the promoter of *Snrpn* and subsequent suppression of gene expression without any alterations in 5hmC distribution (Montalban-Loro et al., 2019).

## TET Proteins in Immune Cell Development and Disease

**Figure 3:**
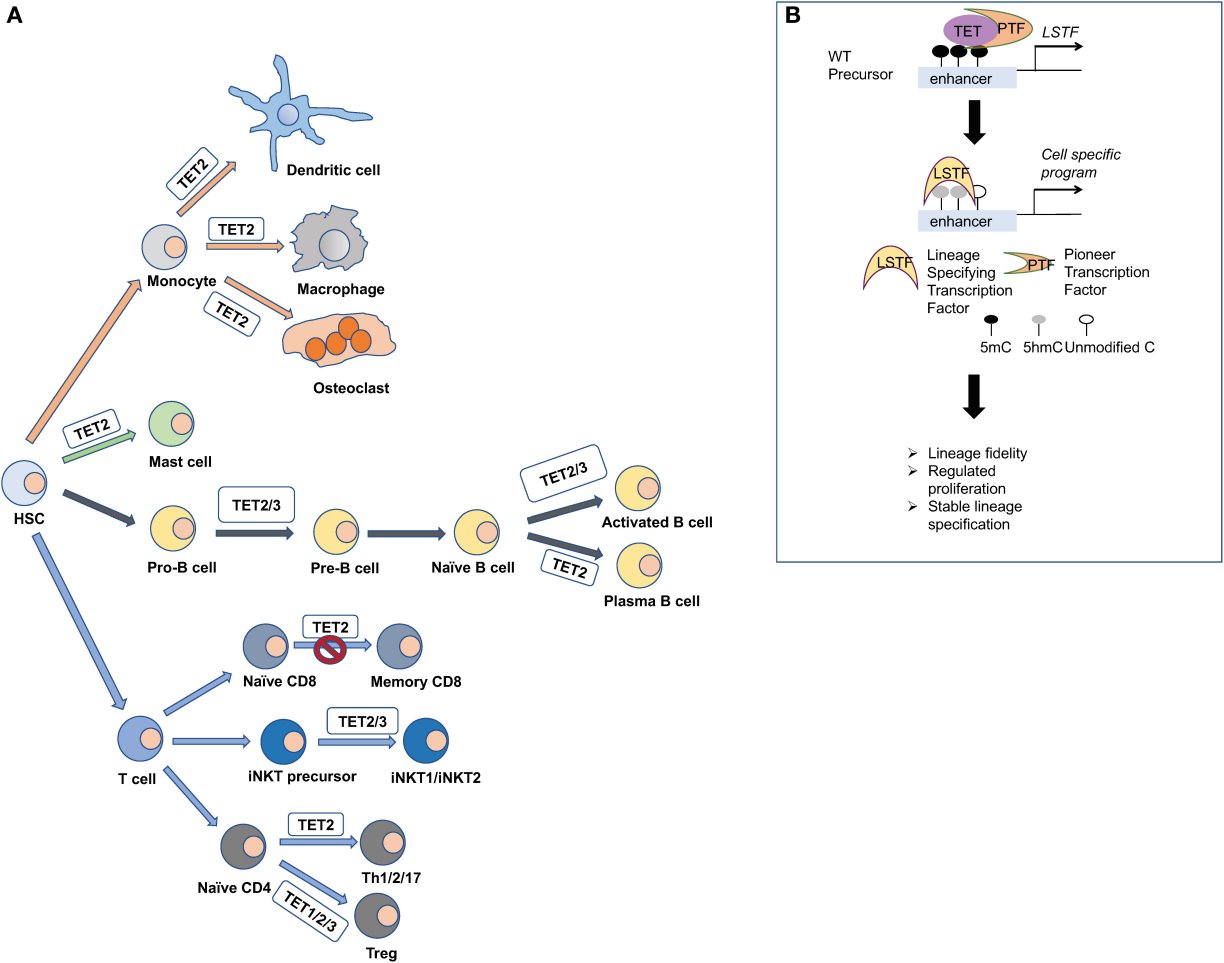
TET proteins orchestrate the differentiation of immune cells. **(A)** Hematopoietic stem cells give rise to the various lineages of our immune system. Mice that are deficient for TET proteins have been used to explore their impact in immune cell development. TET1, TET2, and TET3 have been shown to regulate the methylation status of FOXP3, and they impact the stability of the regulatory T cells (Treg). TET2 and TET3 regulate the iNKT cell lineage specification and are critical for NKT1 and NKT2 cell differentiation. TET2 regulates the formation of memory and effector CD8 cells upon viral infection. During B cell differentiation, TET2 and TET3 orchestrate B cell maturation and function. TET2 also regulates mast cell differentiation and function in both a catalytic-dependent and -independent manner. Moreover, TET2 regulates the function of monocytic populations such as dendritic cells, macrophages, and osteoclasts. **(B)** Mechanistically, TET proteins are recruited by pioneer transcription factors (PTF) at cell-specific enhancers to oxidize 5mC and induce the expression of lineage specifying transcription factors (LSTF). Then, the LSTF execute their cell-specific gene expression program and shape cell identity.

Consistent with their multifaceted regulatory roles, TET proteins have been implicated in various developmental procedures in immune cells (Tsagaratou et al., 2017a; Lio and Rao, 2019) (Figure 3). However, while the implication of TET proteins in DNA demethylation is well-established, the full spectrum of mechanisms that regulate TET proteins in immune cells is yet to be revealed. Immune cell development is a well-characterized process during which progenitor cells, committed in a given pathway of differentiation, give rise to progeny cells (Cumano et al., 2019). This process of differentiation and lineage commitment is irreversible under physiological conditions (Cumano et al., 2019). However, during tumorigenesis, cells de-differentiate or transdifferentiate, frequently resulting in novel cell types that represent a mix of multiple lineages (Le Magnen et al., 2018). Strikingly, TET loss-of-function is strongly associated with hematological malignancies (Cimmino et al., 2011; Shih et al., 2012; Ficz and Gribben, 2014; Huang and Rao, 2014; Ko et al., 2015): *TET2* loss-of-function mutations are frequently observed in myelodysplastic syndromes and myeloid malignancies (Ko et al., 2010; Cimmino et al., 2011; Shih et al., 2012) as well as in certain peripheral T-cell lymphomas (PTCL) (Couronne et al., 2012; Palomero et al., 2014; Sakata-Yanagimoto et al., 2014), which are a heterogeneous and poorly understood group of aggressive non-Hodgkin lymphomas that are resistant to conventional therapies (Armitage, 2012). Loss-of-function studies in mice have been instrumental in unraveling the biological roles of TET proteins in immune cell development, function, and malignant transformation.

### TET Proteins and T-Cell Lineage Fate

In T-cells, loss of TET proteins results in a variety of developmental phenotypes that can compromise immune function or trigger malignant transformation. During the process of T-cell development and lineage specification, 5hmC exhibits dynamic enrichment as precursor cells differentiate to progeny (Tsagaratou et al., 2014). In the thymus, 5hmC is increased in the gene body of lineage-specifying transcription factors such as ThPOK, the factor that seals the fate of CD4 lineage, and RUNX3, the factor that determines the CD8 cell lineage, specifically at the cell stage at which these factors are expressed (Tsagaratou et al., 2014). It has been reported that murine T-cells that lack TET2 exhibit compromised differentiation toward helper lineages such as Th1 and Th17 (Ichiyama et al., 2015) in addition to reduced expression of cytokines such as IFNγ and IL-10 (Ichiyama et al., 2015). Loss of TET2 results in increased representation of CD8 memory T-cells (Carty et al., 2018). *In vitro* polarization of human, naïve CD4 T-cells toward helper lineages demonstrates that DNA demethylation and 5hmC remodeling across the genome occur early after activation and before any differentiation (Nestor et al., 2016; Monticelli, 2019; Vincenzetti et al., 2019). Studies using T-cell polarization cultures suggest that 5hmC plays an important role in directing the specification toward helper lineages but is not necessary for expansion (Vincenzetti et al., 2019).

The most profound phenotypes in T-cells have been observed upon concomitant deletion of at least two TET members, suggesting functional redundancy (Tsagaratou et al., 2017a; Lio and Rao, 2019). Deletion of TET2 and TET3 at the DP cell stage using CD4-cre mice results in a striking increase of the iNKT cell lineage (Tsagaratou et al., 2017b; Tsagaratou, 2018). Furthermore, the *Tet2/3* DKO iNKT cells show lineage skewing in addition to an increase in the NKT17 cell lineage (Tsagaratou et al., 2017b; Tsagaratou, 2019). Moreover, the NKT1 cells are functionally impaired and maintain high expression of stemness genes, such as *Lef1, Lmo1*, and *Myc* (Tsagaratou et al., 2017b), that are usually expressed in earlier stages of iNKT cell development. In this setting, TET proteins regulate the deposition of 5hmC across the gene body of *Tbx21* and *Zbtb7b*, which encode for the lineage specifying factors T-bet and ThPOK, respectively. Upregulation of T-bet is critical for establishing the NKT1 cell fate. At a genome-wide level, loss of TET proteins in iNKT cells does not result in massive DNA demethylation, but rather exerts a focal impact on differential DNA methylation (Tsagaratou et al., 2017b).

These *Tet2/3* DKO iNKT cells can mediate a TCR driven, transmissible T-cell lymphoma upon transfer to fully immunocompetent congenic recipients (Tsagaratou et al., 2017b). However, transplantation of the *Tet2/3* DKO iNKT cells to Cd1dKO mice—incapable of expressing CD1d and presenting antigens to iNKT cells—fails to recapitulate the expansion, indicating an instrumental role of TCR activation in the expansion process. Further analysis revealed that the *Tet2/3* DKO iNKTs that have been transplanted and expanded in congenic recipients show accumulation of DNA breaks and R-loops, signifying that they are undergoing replication stress (Lopez-Moyado et al., 2019).

### TET Proteins and Stability of Regulatory T-Cells

*Tet2*-deficient mice show reduced generation of regulatory T-cells (Tregs) (Nair et al., 2016). Indeed, loss of TET1 and TET2 significantly impairs Tregs by compromising the demethylation of the CNS2 locus of FOXP3: both TET1 and TET2 can bind to this locus (Yang et al., 2015). Concomitant loss of TET2 and TET3 at the DP cell stage using CD4-cre mice exerts more severe impact on the stability of the Foxp3 expression due to aberrant methylation of the CNS2 locus (Yue et al., 2016). Enhancing catalytic activity of TET proteins with vitamin C can promote *in vitro* demethylation of the CNS2 locus, resulting in the generation of induced Tregs (iTregs) with superior stability compared to iTregs generated in vitamin C-absent culture (Xue et al., 2016). This observation is valid for both murine and human iTregs (Yue et al., 2016). Deleting TET2 and TET3 specifically at Tregs using Foxp3-cre mice not only compromises the stability of the Foxp3 lineage, but it also results in gain of effector function and aberrant hyperactivation of the *Tet2/3* deficient Tregs; this leads to increased inflammation and ultimately death of the mice (Nakatsukasa et al., 2019; Yue et al., 2019). In addition, hydrogen sulfide (H2S) was found to induce expression of TET1 and TET2 by regulating binding of nuclear transcription factor y subunit B (NFYB) in the promoters of *Tet1* and *Tet2* (Yang et al., 2015). H2S deficiency results in reduced expression of TET1 and TET2 in T-cells and impaired Treg differentiation due to hypermethylation of the CNS2 locus (Yang et al., 2015). In Tregs, TET recruitment to the CNS2 locus is mediated by transcription factor STAT4 (Yang et al., 2015).

Interestingly, altered metabolism in T-cells can impact the methylation status of regulatory loci in genes that encode for lineage-specifying transcription factors, ultimately affecting the lineage choice of T-cells. Indeed, it has been reported that the glutamate oxalo-acetate transaminase 1 (GOT1) is preferentially expressed in differentiating Th17 cells and catalyzes the conversion of glutamate into a-ketoglutaric acid, resulting in increased levels of 2-hydroxyglutarate (2-HG) (Xu et al., 2017). 2-HG inhibits TET catalytic activity, resulting in increased methylation and reduced expression of FOXP3, the key transcription factor that shapes the Treg lineage (Xu et al., 2017).

### TET Proteins in B-Cell Development and Disease

TET-dependent 5hmC deposition and DNA demethylation are important sources of epigenetic regulation in B-cell development. TET protein expression is regulated dynamically throughout B- lymphopoiesis in humans and mice. Expression of TET1 is drastically reduced in pro-B-cells (Cimmino et al., 2015), while expression of TET2 and TET3 increases progressively over B-cell maturation and during activation (Schoeler et al., 2019). Tet-mediated 5hmC accumulation in B-cells was shown to occur within gene bodies (Cimmino et al., 2015; Schoeler et al., 2019) and at enhancer regions (Lio et al., 2016; Orlanski et al., 2016), additionally correlating with H3K4me1 histone modifications and increased transcriptional activity (Lio et al., 2016; Orlanski et al., 2016). Loss of TET1 in hematopoietic stem cells promotes differentiation with a lymphoid bias (Cimmino et al., 2015). *In vitro* analysis of *Tet1*^−/−^ cells resulted in more self-renewing pro-B-cell colonies compared to pre-B-cell colonies (Cimmino et al., 2015). These proliferating TET1-deficient pro-B-cells show increased accumulation of DNA breaks as attested by increased staining for γH2Ax (Cimmino et al., 2015). In the long-term, germline deletion of TET1 results in lymphocytosis in TET1 deficient mice by 18–24 months of age (Cimmino et al., 2015). Interestingly, transplantation of TET1-deficient cells isolated from the spleen or the lymph nodes of the TET1KO mice to congenic recipients could fully recapitulate the disease within 12 weeks, thereby establishing TET1 as a tumor suppressor of B-cell malignancy (Cimmino et al., 2015).

TET2 is frequently mutated in diffuse large B-cell lymphoma (DLBCL) (Reddy et al., 2017). Deletion of *Tet2* using either Vav-cre or CD19-cre resulted in germinal center hyperplasia (Dominguez et al., 2018). Loss of TET2-mediated 5hmC deposition in enhancer regions of genes involved in exiting the germinal center (GC) reaction also correlated with reduced transcriptional activity (Dominguez et al., 2018). TET2 was instrumental for class switch recombination (CSR) and affinity maturation. TET2 deficient GC B-cells showed a defect in plasma cell differentiation (Dominguez et al., 2018). Moreover, loss of TET2 resulted in downregulation of *Prdm1*, which encodes for BLIMP1. Interestingly, reconstitution of the expression of BLIMP1 in *Tet2-*deficient naïve B-cells by retroviral transduction could rescue the differentiation defects of *Tet2* KO cells (Dominguez et al., 2018). Collectively, these data establish TET2 as a tumor suppressor of B-cell lymphomas (Dominguez et al., 2018).

Interestingly, TET2 mutations in human DLBCLs result in altered gene expression, reminiscent of the *Tet2*-deficient GC gene signature (Dominguez et al., 2018). Comparative analysis of gene expression profiles revealed strong similarities with cases that had mutations in the histone acetyltransferase CREBBP (Dominguez et al., 2018). Thus, TET2 and CREBBP could potentially collaborate to regulate enhancer activation by generating 5hmC and H3K27Ac (Dominguez et al., 2018).

Consistent with observations in T-cells, simultaneous deletion of TET2 and TET3 resulted in more severe B-cell phenotypes. During bone marrow development, ablation of TET2 and TET3 in B-cells using the Mb1-cre mice inhibited B-cell maturation; *Tet2/3* DKO mice exhibited an accumulation of pro- and pre-B-cells, while the mature B-cells were significantly decreased (Lio et al., 2016; Orlanski et al., 2016). TET2 and TET3 were shown to play a critical role in DNA demethylation of the enhancers of Igk light chains (Lio et al., 2016; Orlanski et al., 2016). The recruitment of TET proteins at the enhancer was mediated by the pioneer transcription factor PU.1 (Lio et al., 2016). In addition, TET2 and TET3 regulate the expression of IRF4 and IRF8 that are involved in Igk rearrangement (Lio et al., 2016). Addition of ascorbic acid promotes the differentiation of germinal center B cells to plasma cells both *in vitro* and *in vivo* (Qi et al., 2020). This is achieved by enhancing TET2 and TET3 catalytic activity to demethylate enhancers that control the expression of *Prdm1* (Qi et al., 2020).

TET2 and TET3 proteins regulate somatic hypermutation (SHM) and CSR through Tet-dependent upregulation of Activation Induced Deaminase (AID) in activated B-cells (Lio et al., 2019a; Schoeler et al., 2019). These studies showed that TET proteins were recruited to two sites within the AID super-enhancer, *TetE1* and *TetE2* (Lio et al., 2019a), by the basic leucine zipper transcription factor, ATF-like (BATF) (Lio et al., 2019a; Schoeler et al., 2019).

### TET Proteins in Innate Cell Development and Disease

**Figure 4:**
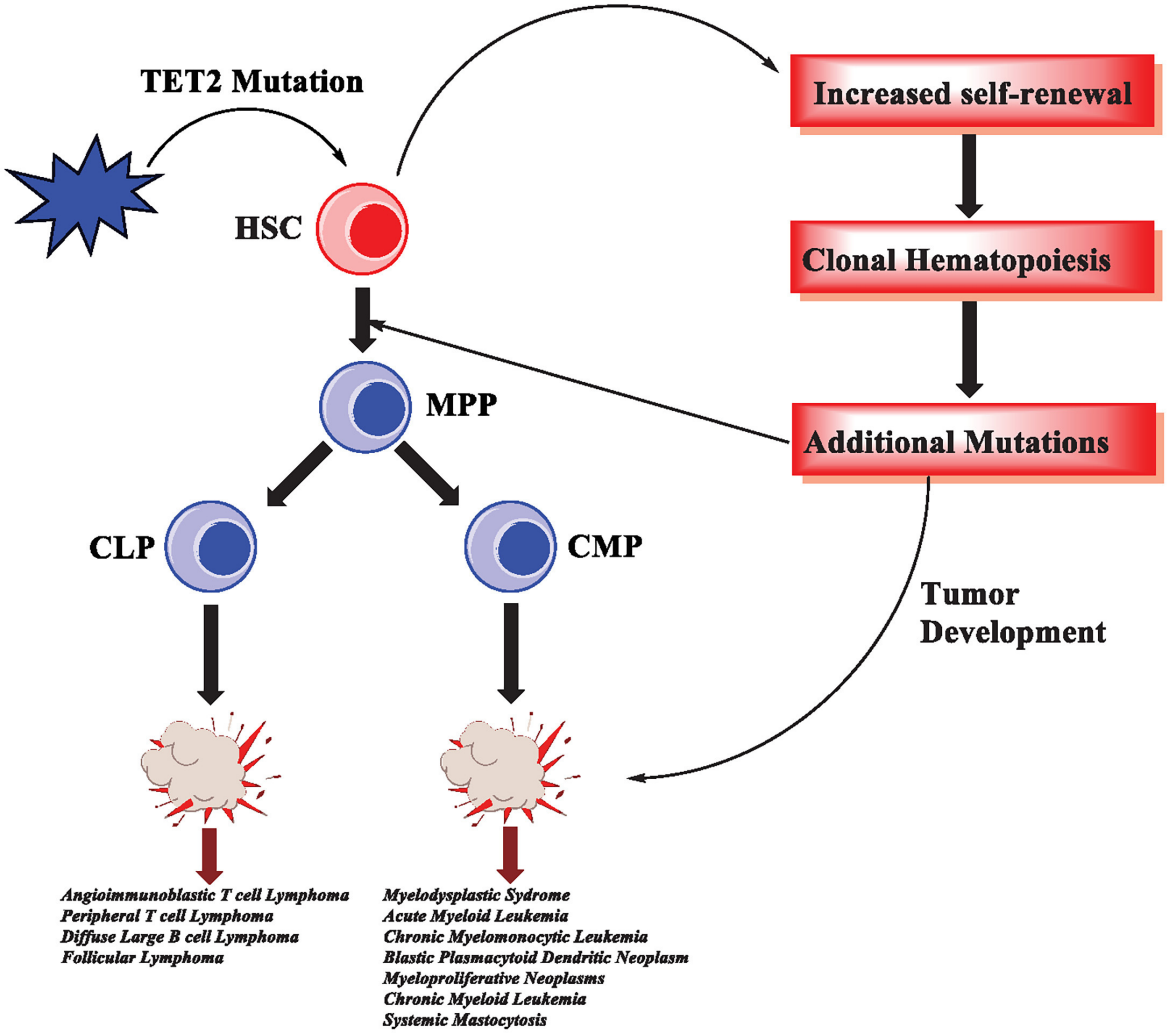
TET2 mutations in hematological malignancies. In hematopoietic stem cells, TET2 mutations are an early event that results in increased self-renewal and subsequently clonal hematopoiesis. As the cells acquire additional mutations, malignant transformation and tumorigenesis occur. TET2 mutations have been reported in various hematological malignancies, affecting myeloid cells as well as T-cell and B-cell lymphomas.

Loss of TET2 in hematopoietic stem cells (HSCs) results in increased stem cell self-renewal, increased number of progenitor cells, and skewed development toward the monocyte/macrophage lineage (Ko et al., 2011; Moran-Crusio et al., 2011) (Figure 4). Additionally, TET2 loss impacts mast cell differentiation and cytokine production as well as proliferation (Montagner et al., 2016). Interestingly, other TET proteins can compensate for altered cell differentiation, suggesting functional redundancy. However, proliferation is exclusively TET2-dependent and independent of its catalytic activity (Montagner et al., 2016). The precise mechanism by which TET2 non-catalytic function is achieved remains unknown, but a plausible scenario is that TET2 could form a complex with other proteins involved in regulating gene expression.

Differentiation of monocytes to osteoclasts is characterized by dynamic changes in DNA methylation (de la Rica et al., 2013). Genomic regions that exhibit changes in DNA methylation during osteoclastogenesis were enriched for PU.1, NF-κB, and AP-1 DNA binding motifs (de la Rica et al., 2013). PU.1 motifs were highly enriched in both hypo- and hyper-methylated regions; it was shown that PU.1 could interact with both TET2 and DNMT3b, thus playing a critical role in recruiting these proteins across the genome to regulate osteoclastogenesis (de la Rica et al., 2013). In addition, differentiation of peripheral blood mononuclear cells (PBMCs) to macrophages and osteoclasts revealed that both cell types exhibit similar dynamic changes of DNA methylation and hydroxymethylation (Garcia-Gomez et al., 2017). However, TET2 and TDG exert a dual function to establish the distinct phenotypes of macrophages and osteoclasts. TET2 further oxidizes 5hmC to oxi-mCs, followed by TDG mediating the generation of unmodified C (Garcia-Gomez et al., 2017). In addition, TET2 can mediate the recruitment of the H3K4 histone methyltransferase SETD1A to promote histone methylation (H3K4me3) at genes that are specifically expressed in osteoclasts (Garcia-Gomez et al., 2017). Similarly, *in vitro* differentiation of human monocytes to dendritic cells requires TET2-dependent DNA demethylation (Klug et al., 2013).

Interestingly, *Tet2* haploinsufficiency contributes to transformation *in vivo*, consistent with the fact that *Tet2* monoallelic loss is an important pathogenic event in myeloid malignancies (Moran-Crusio et al., 2011). Although TET2 mutations can lead to malignancies, they are often an early event in a series of mutations (Huang and Rao, 2014; Rasmussen and Helin, 2016) (Figure 4). TET2 mutations can lead to clonal hematopoiesis (CH), a physiological state in which a specific lineage, or clone, of cells expands at a greater rate than other lineages (Challen and Goodell, 2020). Importantly, acute loss of TET proteins using a system of inducible deletion in mice resulted in the rapid emergence of aggressive myeloid leukemia (An et al., 2015).

**Figure 5:**
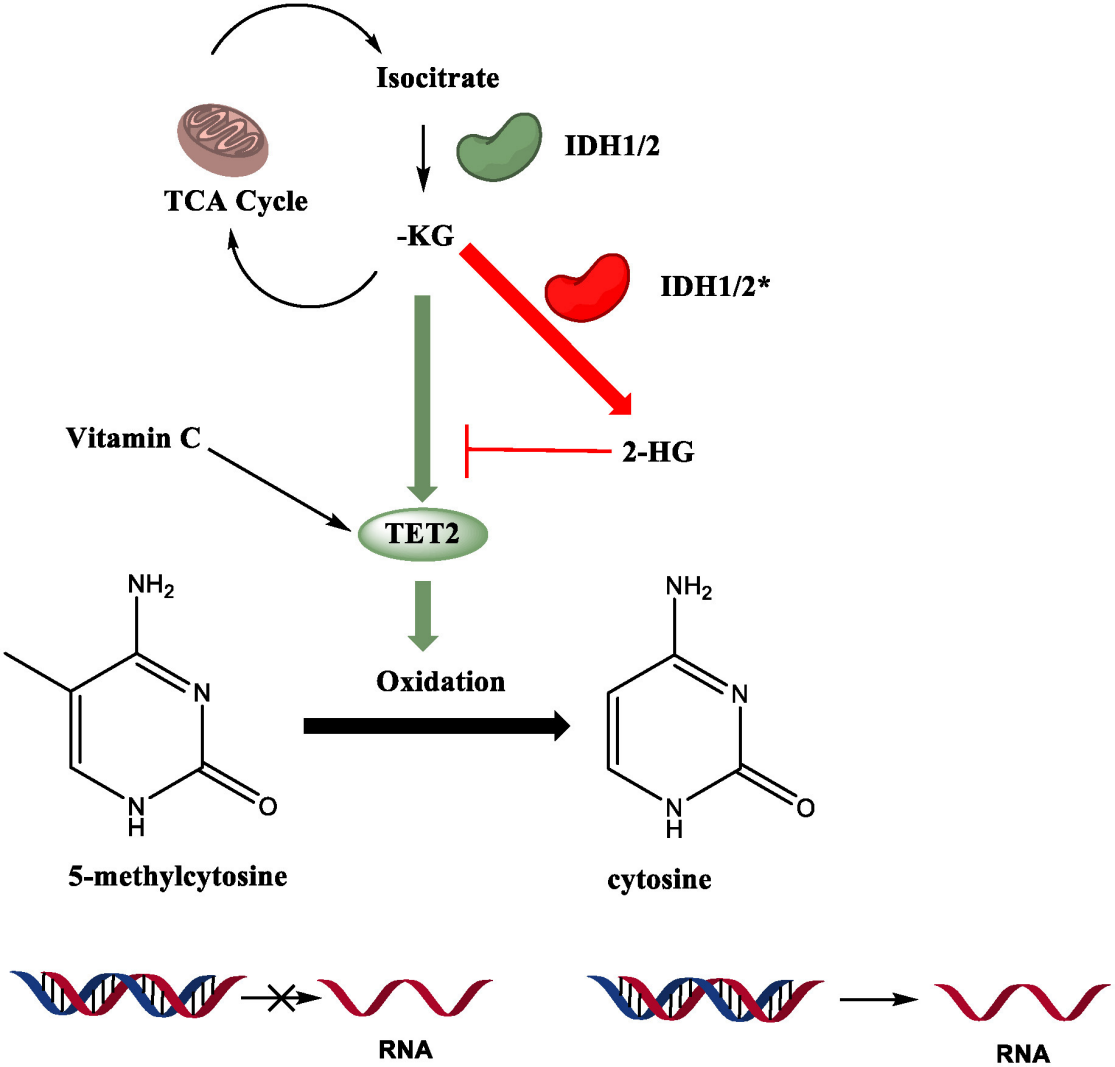
Regulatory mechanisms of TET enzymatic activity. TET2 catalytic activity is Fe (II), alpha-ketoglutarate (α-KG), and oxygen dependent. Mutations in isocitrate dehydrogenase 1 and 2 (IDH), an enzyme in the TCA cycle that converts isocitrate to α-KG, often are gain-of-function (GOF), allowing the enzyme to produce the oncometabolite 2-hydroxyglutarate (2-HG). 2-HG competitively inhibits binding of α-KG to TET2, compromising its function. Vitamin C can enhance the catalytic activity of TET proteins.

Enhancing the activity of TET proteins with vitamin C (Figure 5) can protect hematopoietic stem cells that have *Tet2* mutations from aberrant proliferation *in vitro* and leukemia progression *in vivo* (Agathocleous et al., 2017; Cimmino et al., 2017; Das et al., 2019) (reviewed in Ang et al., 2018; Cimmino et al., 2018; Yue and Rao, 2020). It has been shown that when *Tet2*^+/−^ or *Tet2*^−/−^ HSCs are cultured *in vitro* in the presence of vitamin C, there is an increase of 5hmC compared to *Tet2*-deficient HSCs that are cultured in the absence of vitamin C (Cimmino et al., 2017). The increased 5hmC levels in Tet2^+/−^ HSCs are due to residual TET2 activity and enhanced catalytic activity of TET3, whereas in *Tet2*^−/−^ HSCs the catalytic activity of TET3 is required to oxidize 5mC to 5hmC. The restoration of 5hmC controls cell proliferation (Cimmino et al., 2017). Similarly, *in vivo* administration of vitamin C in xenograft experiments in mice diminished the proliferation rate of *Tet2*-deficient HSCs and reduced tumorigenesis (Cimmino et al., 2017). These findings have significant clinical implications (Ang et al., 2018; Cimmino et al., 2018; Yue and Rao, 2020). Patients with hematological malignancies are often vitamin C-deficient. Oral administration of vitamin C in patients with myeloid malignancies who were on treatment with the DNMT inhibitor azacytidine significantly increased the ratio of 5hmC/5mC in their plasma and restored vitamin C concentration to normal levels (Gillberg et al., 2019).

Mutations in TET2 are not the only pathway that leads to dysfunction. As mentioned earlier, its catalytic activity is Fe (II), alpha-ketoglutarate (α-KG), and oxygen dependent (Tahiliani et al., 2009). Vitamin C (ascorbate) enhances TET activity *in vitro* and *in vivo* (Das et al., 2020). Recent studies have shed light on the connections between metabolism and epigenetic modifiers in physiological and pathological conditions (Chisolm and Weinmann, 2018; Lio et al., 2019b). Disruptions in important metabolic pathways also result in disease states. Mutations in isocitrate dehydrogenase 1 and 2 (IDH), an enzyme in the TCA cycle that converts isocitrate to α-KG, often are gain-of-function (GOF), allowing the enzyme to produce the oncometabolite 2-hydroxyglutarate (2-HG). 2-HG competitively inhibits binding of α-KG to TET2, severely impairing its function (Figure 5). It has been shown that IDH1/2 GOF and TET2 LOF mutations show similar phenotypes in mouse models, with reduced genome-wide 5hmC levels and dysregulated HSC differentiation (Figueroa et al., 2010; Moran-Crusio et al., 2011; Lio et al., 2019b). In myeloid neoplasms, TET2 and IDH1/2 mutations are usually mutually exclusive (Figueroa et al., 2010; Shih et al., 2015; Inoue et al., 2016), but they are often paired together in Angioimmunoblastic T-cell Lymphoma (AITL) (Wang et al., 2015; Cortes and Palomero, 2020). This indicates that there are some differing oncogenic mechanisms at play even given the phenotypic similarities of the two mutations.

Importantly, loss of TET2 can affect the inflammatory response. In fact, TET2 has been implicated in repression of the proinflammatory cytokine interleukin 6 (IL-6), both in macrophages and dendritic cells (Zhang et al., 2015). This is achieved by TET2 interaction with Iκbζ, which permits binding to the *IL6* promoter. Subsequently, TET2, independently of its DNA demethylation activity, mediates the recruitment of the histone deacetylase 2 (HDAC2) to repress *IL6* expression (Zhang et al., 2015). As a result, *Tet2*^−^deficient mice are more susceptible to endotoxin-induced septic shock, induced by administration of lipopolysaccharide (LPS), and colitis compared to control mice, due to exacerbated inflammation (Zhang et al., 2015). Notably, *Tet2*-deficient tumor infiltrating macrophages exhibit defective immunosuppressive capacity in a mouse melanoma model as a result of altered cytokine expression profile (Pan et al., 2017).

It has been reported that CH can result in a 30–40% increased mortality risk unrelated to blood cancers but instead attributed to higher cardiovascular mortality from coronary heart disease and ischemic stroke (Jaiswal et al., 2014; Fuster and Walsh, 2018). Further studies revealed a causal link between TET2 mutations in hematopoietic stem cells, increased inflammation, and atherosclerosis. Competitive transfer of *Tet2*-deficient bone marrow cells resulted in enlarged atherosclerotic lesions in irradiated, atherosclerosis-prone mice that are deficient for low-density lipoprotein receptor (*Ldrl*^−/−^) (Fuster et al., 2017; Jaiswal et al., 2017). *Tet2*-deficient macrophages secreted increased amounts of the cytokine IL-1β in a NLRP3 inflammasome-dependent manner (Fuster et al., 2017). Inhibition of NLRP3 provided enhanced protection from atherosclerosis preferentially to the *Ldrl*^−/−^ mice that had received *Tet2*-deficient bone marrow cells (Fuster et al., 2017).

Similarly, in experimental heart failure mouse models, hematopoietic *Tet2* deficiency followed by competitive transfer or myeloid-specific *Tet2* deficiency resulted in severely impaired cardiac remodeling, accompanied by an NLRP3 inflammasome-dependent increase in IL-1β (Sano et al., 2018). Adoptive transfer of unfractionated *Tet2*-deficient bone marrow cells in non-irradiated recipients revealed that *Tet2* deficiency alters the phenotype of macrophages present in the heart and promotes cardiomyopathy in steady state conditions in aged mice without pre-existing cardiovascular injury (Wang et al., 2020). Gene expression analysis of *Tet2*-deficient derived macrophages 8 months after transfer revealed that IL1-β was upregulated (Wang et al., 2020). The aged mice showed signs of cardiac dysfunction and increased inflammation (Wang et al., 2020). In addition, competitive transfer of TET2KO bone marrow cells exacerbates insulin resistance in aging and obesity in an IL-1β NLRP3 inflammasome-dependent manner (Fuster et al., 2020). Increased inflammation due to *Tet2* loss has recently been associated with pulmonary arterial hypertension in humans as well as in *Tet2*- deficient mice (Potus et al., 2020).

## Perspectives

### TET Proteins Regulate Focal DNA Demethylation

Various studies using TET-deficient mice demonstrated that loss of TET proteins has only a mild impact on global DNA methylation (An et al., 2015; Cimmino et al., 2015; Tsagaratou et al., 2017b). However, when focusing on regions that are differentially methylated across development, a robust increase in DNA methylation was observed upon TET loss (Tsagaratou et al., 2017b). This observation is consistent with the report that only 21.8% of autosomal CpGs exhibit dynamic changes in their methylation status across development (Ziller et al., 2013). This primarily occurs in loci genomically distant from the TSS (Ziller et al., 2013). Loss of TET proteins contributed to increased DNA methylation even in regulatory areas with high methylation levels in T-cell subsets, suggesting that TET proteins compete with DNMTs to avoid aberrant hypermethylation (Tsagaratou et al., 2017b). Maintaining a certain threshold of DNA methylation and/or generating 5hmC could stabilize the enhancers in a poised state, allowing the rapid initiation of gene expression at subsequent developmental stages or following certain environmental cues. This concomitant existence of two opposing epigenetic marks is reminiscent of the poised bivalent promoters that have been extensively described mainly in embryonic stem cells and are characterized by coexistence of the H3K4me3 histone mark, which positively correlates with gene expression, and the suppressing mark H3K27me3 (Bernstein et al., 2006).

The focal activity of TET proteins in DNA demethylation strongly suggests that TET proteins are recruited and targeted to the DNA *via* transcription factors to regulate the DNA demethylation of regulatory elements that control the expression of key genes involved in the cell-specific program of a given immune cell. Indeed, in regulatory T-cells, members of the STAT family act as pioneer transcription factors that exert TET recruitment at enhancers (Yang et al., 2015). In B-cells, PU.1, EBF1, and BATF can mediate TET recruitment to regulatory elements (Lio et al., 2016, 2019a). Open chromatin conformation as well as chromatin accessibility correlates with increased 5hmC levels across a variety of leukocytes (Lio et al., 2016; Tsagaratou et al., 2017b). TET proteins affect TF binding at regulatory elements, including enhancers, by virtue of their cell type-specific binding motifs and role in modifying chromatin accessibility (Rasmussen et al., 2019).

### TET Proteins and Lineage Specification

TET proteins play a critical role in regulating lineage specification of various cell types (Tsagaratou et al., 2017a; Wu and Zhang, 2017). For instance, TET proteins deposit intragenic 5hmC in *Zbtb7b* and *Tbx21*, genes that produce the crucial lineage-specifying factors of T-cell differentiation: ThPOK and RUNX3, respectively. 5hmC enrichment slowly decreases over time upon commitment to a given cell fate (Tsagaratou et al., 2014).

Importantly, TET protein loss results in abnormal development, failure to progress beyond precursor cell stages, and unregulated cell division (Cimmino et al., 2015; Lio et al., 2016; Orlanski et al., 2016; Tsagaratou et al., 2017b). Investigation of TET loss in mutant mice indicated that malignant transformation occurs due to maintenance of a stemness gene expression program instead of commitment to a lineage-specific program.

In addition, TET proteins are instrumental in safeguarding stability of gene expression, preventing de-differentiation of cells. For instance, TET proteins prevent aberrant methylation of regulatory elements to stabilize the expression of the Treg lineage-specifying factor FOXP3. During thymic development, TET1 and TET3 can regulate the cytosine methylation status of enhancers that permit *Cd4* gene expression at later stages in the periphery (Issuree et al., 2018). Presumably, deposition of 5hmC in enhancers can prime these regulatory elements to become fully activated and promote gene expression at subsequent developmental stages.

### TET Proteins and Functional Redundancy

Analysis of various mouse models strongly suggests that TET proteins exhibit redundancy. For example, development proceeds normally in many cases upon deletion of a single TET protein. It seems that TET proteins act in complement to regulate enhancers and lineage-specifying TFs, leading to activation of a cell-specific gene expression program. In addition, mice that lack a single TET protein develop cancer slowly over the course of several years (Cimmino et al., 2015; Lio et al., 2019b). However, simultaneous deletion of two or more TET proteins results in rapid, malignant transformation of a gamut of immune cell lineages (An et al., 2015; Zhao et al., 2015; Lio et al., 2016; Tsagaratou et al., 2017b).

## Future Directions

Since TET proteins are recruited at specific genomic loci by interacting partners, it is critical to unveil the cell-specific interactome of TET proteins that will allow us to gain appreciation of the full spectrum of TET-regulated cell properties. We do anticipate that these interactions will reveal novel, unexpected roles of TET proteins in immune cell development that extend beyond the regulation of DNA demethylation. For example, until recently, it was thought that only mutations in the catalytic region of TET2 could induce oncogenic transformations. However, recent studies have shown that TET2 knockout mice and TET2 mutant mice (with a mutation rendering the catalytic domain non-functional) produce different disease states (Ito et al., 2019). The former resulted in both myeloid and lymphoid malignancies, while the latter produced primarily myeloid malignancies (Ito et al., 2019). This suggests that TET2 has roles as a tumor suppressor independent of its catalytic function. Further investigation is needed to identify its other physiological functions that safeguard the proper differentiation and proliferation of cells. Along these lines, we anticipate that TET1 and TET3 might also exert catalytic-independent functions in the context of immune cell differentiation and function.

An additional future direction of research that will allow us to fully understand the regulatory impact of TET proteins on gene expression is the fact that multiple loci affected by TET proteins are enhancers; thus, it is challenging to qualify the genes that are directly affected by TET loss (Tsagaratou et al., 2017b). It is now largely accepted that enhancers can regulate genes that are located far across the genome. Methods such as Hi-C (Lieberman-Aiden et al., 2009; Rao et al., 2014), ChiA-PET (Fullwood et al., 2009), and HiChIP (Mumbach et al., 2016) not only are expensive ways to assess genome-wide topological associations but also, in many cases, are not easily adjustable to the small numbers of primary cells that can be purified. Future work that can precisely determine the genes comprising the TET regulome will elucidate the causal mechanisms underlying the abnormal immune cell phenotypes present in biological systems lacking TET proteins.

After identifying potential regulatory elements and assigning them to genes that they might regulate, it is critical to confirm experimentally if these enhancers are instrumental for gene expression. Novel genome editing technologies can be employed to test enhancer activity, such as clustered regularly interspaced short palindromic repeats (CRISPR/Cas9) (Catarino and Stark, 2018). Briefly, CRISPR/Cas9 creates double-stranded breaks in target DNA sequences that are specified by sequence complementary guide RNAs (Jinek et al., 2012).

## Conclusions

To conclude, in the last decade we have witnessed significant progress in our understanding of the biology of TET proteins. Besides the well-established enzymatic function of TET proteins that contributes to DNA demethylation, we have started to appreciate additional roles that these proteins assume to regulate gene expression and establish cell identity. As we move forward, it is critical to dissect the unique versus the shared functions of TET proteins, unravel the cell-specific interactome of each TET protein, and decipher the regulatory elements that they control. Ultimately, by harnessing TET enzymatic and non-enzymatic activity, we hope to be able to epigenetically reprogram cells, preventing their hyperproliferation and malignant transformation that ultimately results in tumorigenesis. Moreover, deciphering the mechanisms by which TET2 loss and clonal hematopoiesis result in increased inflammation and age-related cardiovascular diseases can pave the way for therapeutic intervention.

## Author Contributions

NJT and AT wrote the manuscript with contributions from DB, LFC, and FJW. NJT and LFC prepared the figures with input from AT. All authors approved the submitted manuscript.

## Conflict of Interest

The authors declare that the research was conducted in the absence of any commercial or financial relationships that could be construed as a potential conflict of interest.
